# P_II_ protein is essential for transcriptional regulation of *anf* gene cluster for iron-only nitrogenase in *Rhodopseudomonas palustris*

**DOI:** 10.1128/aem.00465-25

**Published:** 2025-04-10

**Authors:** Yan Zeng, Lingwei Cui, Mengmei Wang, Lu Huang, Mingyue Jiang, Ying Liu, Yongqiang Gao, Yanning Zheng

**Affiliations:** 1 State Key Laboratory of Microbial Resources, Institute of Microbiology, Chinese Academy of Sciences 85387, Beijing, China; 2 College of Life Sciences, University of the Chinese Academy of Sciences 617066 , Beijing, China; 3 Department of Microbiology, Harvard Medical School 1811 , Boston, Massachusetts, USA; University of Nebraska-Lincoln, Lincoln, Nebraska, USA

**Keywords:** Fe-only nitrogenase, gene regulation, P_II_ proteins, photosynthetic bacteria, *Rhodopseudomonas*

## Abstract

In addition to catalyzing the biological nitrogen fixation, iron-only (Fe-only) nitrogenase is also able to reduce carbon dioxide (CO_2_) to formate (HCOO^−^) and methane (CH_4_). AnfA is responsible for the transcriptional activation of the *anf* gene cluster for Fe-only nitrogenase, whose expression is repressed by fixed nitrogen. However, it remains unclear how AnfA is regulated to control the expression of Fe-only nitrogenase. Here, we found that in *Rhodopseudomonas palustris*, P_II_ proteins play a critical role in regulating the expression of Fe-only nitrogenase genes via AnfA. We hypothesize that the deuridylylated P_II_ protein GlnK1, which was upregulated in the presence of ammonium (NH_4_^+^), could inhibit AnfA activity by forming a potential AnfA-GlnK1 complex. This likely serves as a fail-safe mechanism to prevent *R. palustris* from expressing Fe-only nitrogenase when AnfA is accidentally expressed under nitrogen-excess conditions. The uridylylated P_II_ protein GlnK2^UMP^, which was upregulated in response to nitrogen starvation, stimulated the expression of an active AnfA hexamer that further activated the expression of Fe-only nitrogenase under nitrogen-fixing and Mo-free conditions. This study provides new insights into the regulation of Fe-only nitrogenase in *R. palustris*.

**IMPORTANCE:**

The expression and maturation of nitrogenase are tightly regulated by ambient nitrogen levels, which limits the persistence and efficiency of biological nitrogen fixation. This study offers new insights into the regulatory mechanism of AnfA by P_II_ proteins in *Rhodopseudomonas palustris*. Understanding the regulation of AnfA, the transcriptional activator of the Fe-only nitrogenase gene cluster, could provide strategies to better control the expression of iron-only nitrogenase. Nitrogen-fixing bacteria that constitutively express iron-only nitrogenase have the potential to be developed into promising biofertilizers, as their nitrogen-fixing activity is enhanced and independent of molybdenum availability in the soil.

## INTRODUCTION

As one of the essential elements for life, nitrogen plays a critical role in the maintenance and continuation of life on Earth. Since the 1960s, the development and extensive application of synthetic nitrogen fertilizer have significantly disrupted the global nitrogen cycle (1, 2). Currently, around 50% of fertilizers are used for non-leguminous crops such as wheat, rice, and maize, with their nitrogen use efficiency typically lower than 40% (3). The nitrogen loss leads to water eutrophication, causing massive hypoxic zones worldwide. Under anoxic conditions, the potent greenhouse gas nitrous oxide (N_2_O), which has a warming potential 300 times that of carbon dioxide (CO_2_), is produced through denitrification (4). Therefore, it is of great significance to replace traditional chemical nitrogen fertilizers by developing efficient and slow-release N-biofertilizers for non-leguminous crops. The application of N-biofertilizers will effectively reduce the emissions of pollutants and promote the development of sustainable agriculture.

Biological nitrogen fixation is catalyzed by nitrogenase, which can be classified into molybdenum (Mo) nitrogenase, vanadium (V) nitrogenase, and iron (Fe)-only nitrogenase based on the metal composition of the cofactor at the active site (5). Mo is often deficient in bioavailable forms, particularly in acidic soils (6). Therefore, Fe-only nitrogenase that harbors no heterometals has advantages over Mo nitrogenase in farmlands where bioavailable Mo is scarce. *Rhodopseudomonas palustris*, which encodes all three types of nitrogenases, preferentially expresses the Mo nitrogenase, with V nitrogenase and Fe-only nitrogenase serving as alternative nitrogenases. All three nitrogenases are subject to ammonium (NH_4_^+^) inhibition. In addition, Fe-only nitrogenase expression is regulated in response to Mo and V availability. It can only be expressed when no active Mo and V nitrogenases are present (7, 8). Mo-responsive regulation is widely regarded as the primary mechanism governing the differential expression of Mo and Fe-only nitrogenases (9, 10). However, the expression of Fe-only nitrogenase genes is induced by increasing levels of nitrogen starvation in *R. palustris*, rather than by direct Mo regulation (7). Similar behavior has been observed in *Rhodospirillum rubrum*, which produces Fe-only nitrogenase in the presence of Mo when it cannot express an active Mo nitrogenase (11). Furthermore, the activities of all three nitrogenases in *R. palustris* are inhibited via ADP-ribosylation when grown with NH_4_^+^ (12).

Although little is known about the genetic regulation of Fe-only nitrogenase, the regulation of Mo nitrogenase has been well studied in *R. palustris*. P_II_ proteins play a key role in nitrogenase regulation. *R. palustris* encodes three P_II_ proteins: GlnK1, GlnK2, and GlnB. Under nitrogen-fixing conditions, all three P_II_ proteins undergo uridylylation, while under nitrogen-excess conditions, they exist in deuridylylated forms (13). In nitrogen-excess conditions, *R. palustris* predominantly expresses GlnK1 (14). The deuridylylated P_II_ protein is unable to activate the NtrBC two-component system to stimulate the synthesis of NifA, a transcriptional activator for Mo nitrogenase (15). In the meanwhile, the deuridylylated GlnK1 forms an inactive NifA-GlnK1 complex by interacting with NifA (14), further inhibiting the expression of Mo nitrogenase in photosynthetic diazotrophs (15, 16). Under conditions of NH_4_^+^ limitation, *R. palustris* switches to expressing GlnK2 (14). The uridylylated GlnK2 (GlnK2^UMP^), which is incapable of binding to NifA, stimulates the expression of fully active NifA, thereby promoting the expression of Mo nitrogenase (14). The P_II_^UMP^ has been shown to be crucial for NifA activity in *Azospirillum brasilense* and *R. rubrum* (17, 18). Similar to Mo nitrogenase, the expression of Fe-only nitrogenase is also regulated by a σ^54^-dependent transcriptional activator AnfA, which is homologous to NifA (19). However, it remains unclear whether P_II_ proteins interact with AnfA and subsequently regulate the expression of Fe-only nitrogenase.

Here, we explore how P_II_ proteins regulate both the expression and activity of AnfA, providing new insights into the regulation of Fe-only nitrogenase. We found that AnfA was primarily regulated at the transcriptional level by GlnK2 in conditions of nitrogen limitation. We also hypothesized that deuridylylated GlnK1 inhibited AnfA activity by direct interaction under nitrogen-excess conditions, thereby preventing AnfA from stimulating the expression of Fe-only nitrogenase genes.

## RESULTS

### AnfA is subject to both transcriptional and post-translational regulation in *R. palustris*

**Fig 1:**
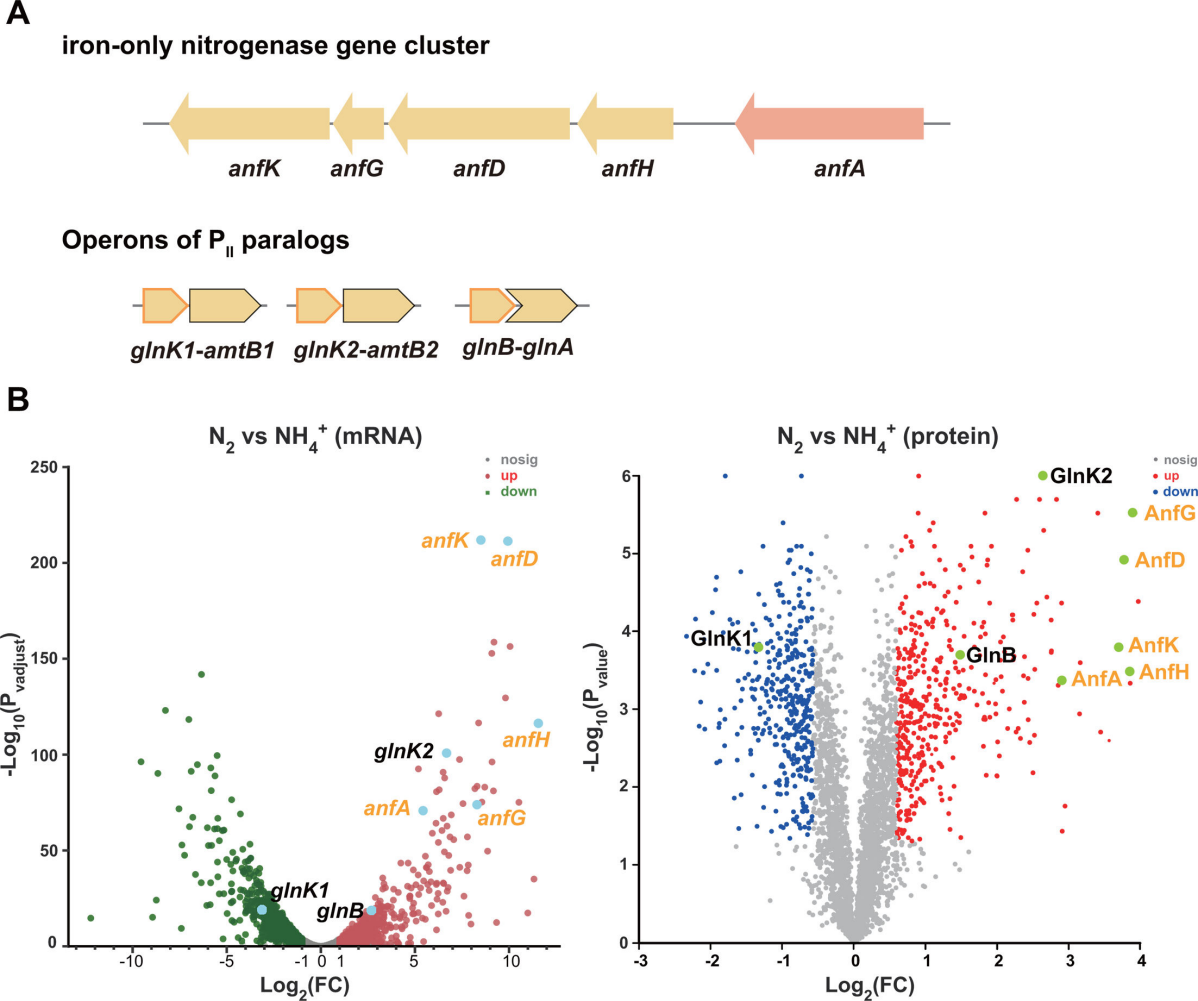
Transcriptomic and proteomic analyses indicate P_II_ proteins may regulate the expression of Fe-only nitrogenase. (**A**) The operons of all the P_II_ genes and the *anf* gene cluster in *R. palustris* CGA009. (**B**) For transcriptome and proteome sequencing, *R. palustris* CGA009 was grown with nitrogen gas (N_2_) as the sole nitrogen source for diazotrophic growth. In contrast, CGA009 grown with NH_4_^+^ for non-diazotrophic growth was used as the control. Mo was removed from both nitrogen-excess (NH_4_^+^) and nitrogen-fixing (N_2_) media. In addition to *anfHDGK* and *anfA* genes coding for Fe-only nitrogenase and its transcriptional activator, the P_II_ genes (*glnK1*, *glnK2*, and *glnB*) and their protein products were also differentially expressed, suggesting that P_II_ proteins may play pivotal roles in Fe-only nitrogenase regulation. The volcano plots were generated by the ggplot2 R package. The thresholds of |log_2_FC| ≥ 1 (FC, fold change) and |log_2_FC| ≥ 0.585 were used to define differentially expressed genes and proteins, respectively.

While the role of P_II_ proteins in the regulation of Mo nitrogenase expression is well understood, the mechanisms underlying the transcriptional activation of the Fe-only nitrogenase gene cluster remain unclear. To explore the role of P_II_ proteins in regulating Fe-only nitrogenase expression, we analyzed the transcriptome (SRA: PRJNA978312) and proteome (iProX: PXD042610) of *R. palustris* CGA009 (wild-type [WT]) under nitrogen-excess (NH_4_^+^) and nitrogen-fixing (N_2_) conditions. Given that active Mo nitrogenase inhibits the expression of Fe-only nitrogenase, Mo was omitted from both nitrogen-excess and nitrogen-fixing media during the cultivation of *R. palustris* CGA009 cells. Under nitrogen-fixing conditions, *R. palustris* CGA009 exhibited significant upregulation of the *anfA* gene at both transcriptional (43.1-fold) and translational (7.5-fold) levels compared to nitrogen-excess conditions (Fig. 1). These results indicate that *anfA* is subject to regulation at the transcriptional level. Additionally, the *glnK1*, *glnK2,* and *glnB* genes encoding P_II_ proteins showed significant differential expression under nitrogen-excess and nitrogen-fixing conditions. *R. palustris* primarily expressed the *glnK1* gene under nitrogen-excess conditions (mRNA, *glnK1:glnK2:glnB* = 18:1:4), while it switched to primarily expressing *glnK2* under nitrogen-fixing conditions (mRNA, *glnK1:glnK2:glnB* = 1:51:12). Given that AnfA directly activates the expression of Fe-only nitrogenase, these differentially expressed P_II_ proteins may have distinct roles in regulating AnfA in response to nitrogen status.

**Fig 2:**
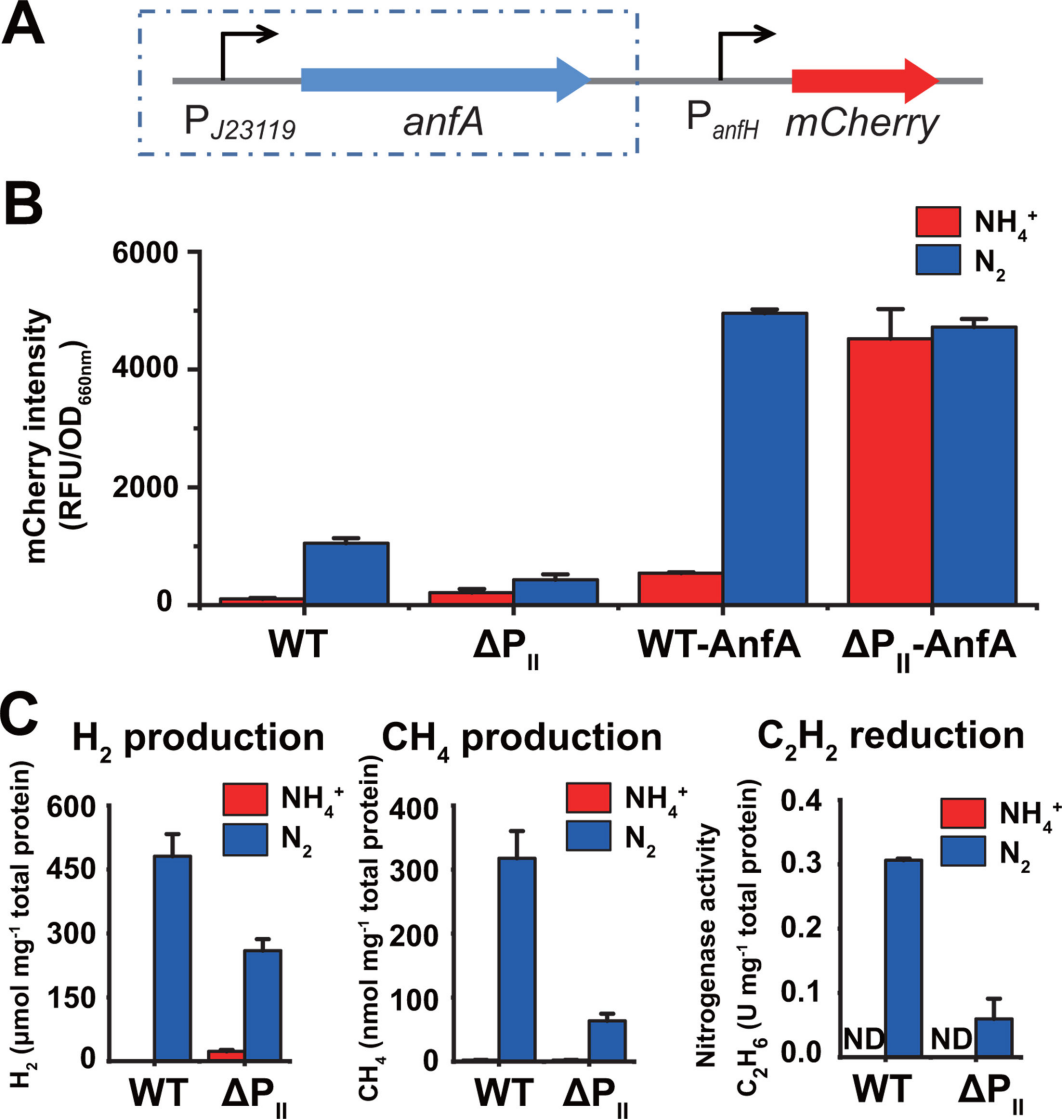
P_II_ proteins are involved in the transcriptional and posttranslational regulation of AnfA. (**A**) An RFP reporter system in plasmid with the *mCherry* gene expressed from the promoter (P*_anfH_*) of Fe-only nitrogenase genes (*anfHDGK*) was created to monitor the Fe-only nitrogenase expression by fluorescence intensity. When necessary, AnfA was overexpressed from a strong constitutive promoter P*_J23119_*. (**B**) The effect of P_II_ proteins on the expression and activity of AnfA. *R. palustris* CGA009-7 (WT), CGA3033-13 (ΔP_II_), CGA009-8 (WT-AnfA), and CGA3033-14 (ΔP_II_-AnfA) strains expressing *mCherry* from P*_anfH_* promoter were grown with NH_4_^+^ (red bars) or N_2_ gas (blue bars) under Mo-free conditions. The fluorescence intensity of mCherry was indicated by the relative fluorescence unit (RFU) normalized to optical density of *R. palustris* at 660 nm (OD_660_). *R. palustris* strains: CGA009-7, GGA009 expressing *mCherry* from P*_anfH_*; CGA009-8, GGA009 expressing *mCherry* from P*_anfH_* and *anfA* from P*_J23119_*; CGA3033-13, a *glnK1 glnK2 glnB* triple deletion mutant (ΔP_II_) expressing *mCherry* from P*_anfH_*; CGA3033-14, ΔP_II_ expressing *mCherry* from P*_anfH_* and *anfA* from P*_J23119_*. (**C**) H_2_/CH_4_ production and C_2_H_2_ reduction were determined by gas chromatography to examine the activity of Fe-only nitrogenase. These data are the average of three independent experiments, and the error bars represent the SD.

To examine the roles of the P_II_ proteins in the regulation of AnfA, we measured the expression of Fe-only nitrogenase in an *R. palustris* ΔP_II_ mutant (14) grown without Mo using the red fluorescent protein (RFP) mCherry (20), which was driven by the P*_anfH_* promoter of the Fe-only nitrogenase gene cluster (Fig. 2A). The *R. palustris* ΔP_II_ mutant was obtained by deleting all three P_II_ genes (*glnK1*, *glnK2,* and *glnB*) as well as two NH_4_^+^ transporter genes (*amtB1* and *amtB2*), which are located in operons with *glnK1* and *glnK2*, respectively. Previous studies have demonstrated that the *amtB1 amtB2* double deletion mutant retains the ability to take up NH_4_^+^ and does not affect the regulation of nitrogenase (21). The ΔP_II_ mutant expressed lower levels of Fe-only nitrogenase under nitrogen-fixing and Mo-free conditions (Fig. 2B), resulting in reduced activity of acetylene (C_2_H_2_) reduction and decreased production of hydrogen (H_2_) and methane (CH_4_) (Fig. 2C). Given that the reduction of C_2_H_2_ to ethane (C_2_H_6_) is a unique property of Fe-only nitrogenase and the C_2_H_2_ to ethylene (C_2_H_4_) activity of Fe-only nitrogenase is just about 1% of the activity of Mo nitrogenase, we determined the activity of Fe-only nitrogenase by measuring C_2_H_6_ instead of C_2_H_4_ to avoid any potential interference from residual Mo nitrogenase activity, which could occur even if trace amounts of Mo are present in the medium. The same as the WT strain, ΔP_II_ mutant grown with NH_4_^+^ expressed no Fe-only nitrogenase by RFP analysis, with no C_2_H_2_ reduction as well as H_2_ and CH_4_ production detected by gas chromatography analysis (Fig. 2C). These data demonstrate that AnfA synthesis is subject to transcriptional regulation by P_II_ proteins.

Subsequently, we overexpressed AnfA in *R. palustris* CGA009 and ΔP_II_ mutant, respectively. The ΔP_II_ mutant expressing AnfA showed significant expression of Fe-only nitrogenase even in the presence of NH_4_^+^ under Mo-free conditions. In contrast, no Fe-only nitrogenase expression was observed in CGA009 expressing AnfA under the same conditions (Fig. 2B). The CGA009 and ΔP_II_ mutant expressing AnfA had comparable expression levels of Fe-only nitrogenase when they were grown under nitrogen-fixing and Mo-free conditions. AnfA showed full activity in the absence of P_II_ proteins. Therefore, AnfA activity is subject to post-translational regulation by P_II_ proteins.

### Deuridylylated P_II_ proteins inhibit the activity of AnfA

Upon examining the expression of GlnK1, GlnK2, and GlnB in response to NH_4_^+^ under Mo-free conditions, a different expression pattern for GlnK1 was observed when compared to GlnK2 and GlnB. GlnK1 was significantly upregulated in NH_4_^+^-grown *R. palustris* cells, while GlnK2 and GlnB were predominantly expressed under nitrogen-fixing conditions (Fig. 1). Therefore, GlnK1, GlnK2, and GlnB may play different roles in modulating the expression of Fe-only nitrogenase.

**Fig 3:**
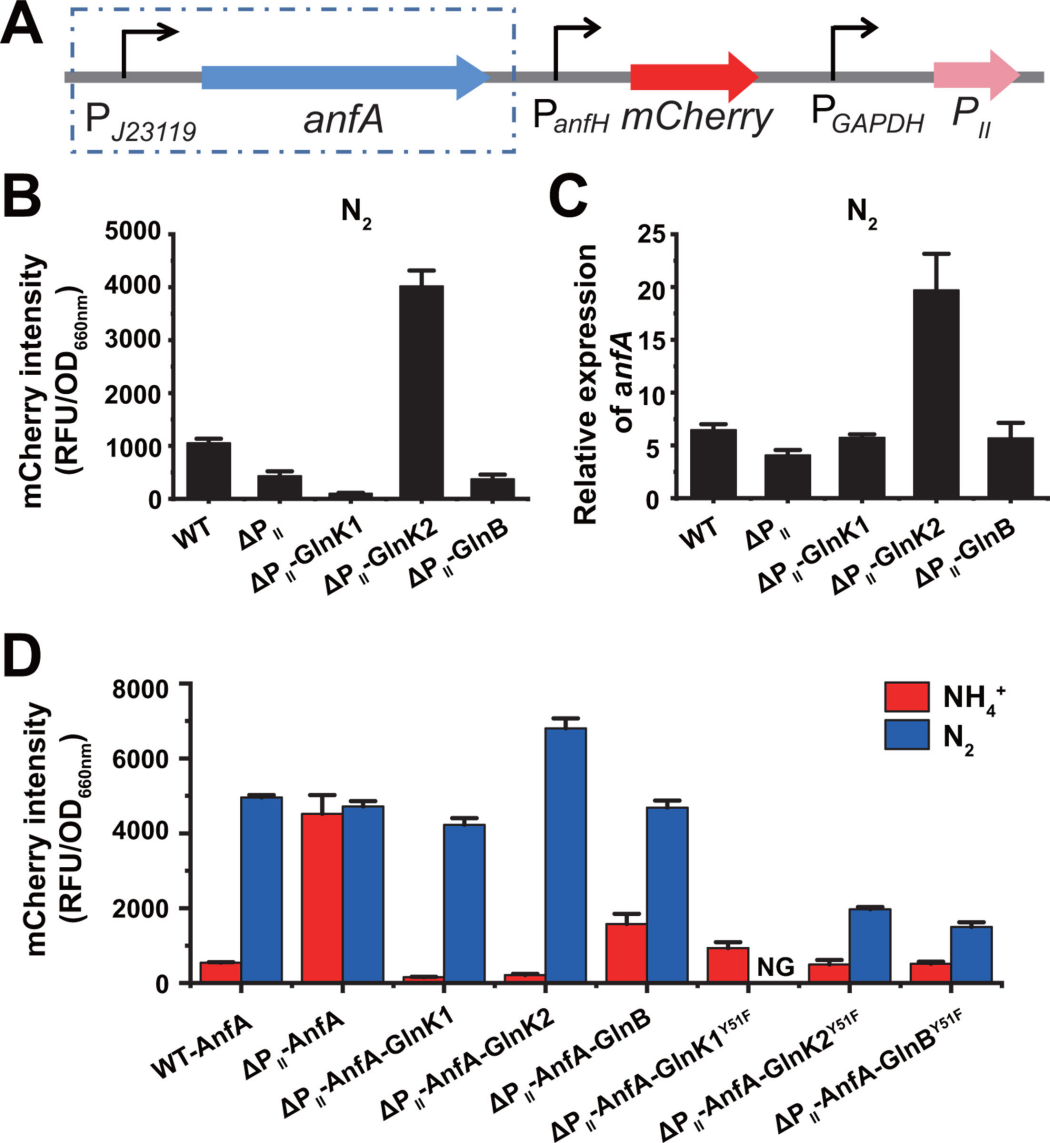
Uridylylated GlnK2 stimulates the expression of *anfA,* and deuridylylated GlnK1 inhibits the activity of AnfA. (**A**) An RFP reporter system was created to examine the influence of P_II_ proteins or their variants P_II_^Y51F^ on the expression of the *anfA* gene and the activity of AnfA protein, respectively. When necessary, AnfA, P_II_, or P_II_^Y51F^ was overexpressed from a strong constitutive promoter (P*_J23119_* or P*_GAPDH_*). (**B and C**) The effect of P_II_ proteins on the activity of AnfA protein (**B**) and the expression of *anfA* gene (**C**). GlnK1, GlnK2, and GlnB were overexpressed in ΔP_II_ mutant grown to the logarithmic phase under nitrogen-fixing and Mo-free conditions. The ΔP_II_ mutant complemented with plasmid carrying the *glnK2* gene (CGA3033-16) greatly enhanced the expression of *anfHDGK* and *anfA* genes, which were determined by fluorescence intensity of RFP reporter and qRT-PCR, respectively. (**D**) Deuridylylated P_II_ proteins inhibit AnfA activity *in vivo*. The deuridylylated GlnK1, GlnK2, GlnB, GlnK1^Y51F^, GlnK2^Y51F^, and GlnB^Y51F^ were expressed in *R. palustris* strains grown with NH_4_^+^ or N_2_ under Mo-free conditions. As the uridylylation site of GlnK1, GlnK2, and GlnB, the amino acid substitution Y51F makes GlnK1, GlnK2, and GlnB unable to be uridylylated even under nitrogen-fixing conditions. NG, no growth. These data are the average of three independent experiments, and the error bars represent the SD.

To investigate how GlnK1, GlnK2, or GlnB influences AnfA activity, we complemented the ΔP_II_ mutant with a plasmid expressing GlnK1, GlnK2, or GlnB (Fig. 3A). Under nitrogen-fixing and Mo-free conditions, P_II_ proteins undergo uridylylation at the Tyr^51^ residue in the T-loop, catalyzed by the bifunctional uridylyltransferase/uridylyl-removing enzyme (GlnD) (14, 22). Complementation of the ΔP_II_ mutant with GlnK2 (GlnK2^UMP^) significantly enhanced the expression of Fe-only nitrogenase compared to both the WT strain and the ΔP_II_ mutant (Fig. 3B and C). In addition, the *glnK1*, *glnK2,* or *glnB* single deletion mutant exhibited phenotypes similar to those of the ΔP_II_ mutant (Fig. S1). These data suggest that uridylylated GlnK2 stimulates the expression of active AnfA. Under nitrogen-excess and Mo-free conditions, complementation of the ΔP_II_ mutant with any of the P_II_ proteins significantly reduced the expression of Fe-only nitrogenase, indicating that AnfA activity is inhibited by P_II_ proteins in the presence of NH_4_^+^. To confirm that the deuridylylated form of the P_II_ protein was responsible for inhibiting AnfA, we generated three P_II_ variants (GlnK1^Y51F^, GlnK2^Y51F^ and GlnB^Y51F^), which are unable to undergo uridylylation. Complementation with the deuridylylated P_II_ variants, particularly GlnK1^Y51F^, resulted in a dramatic reduction in Fe-only nitrogenase expression, regardless of the presence or absence of NH_4_^+^ (Fig. 3D). These data indicate that deuridylylated P_II_ proteins inhibit the activity of AnfA and further prevent the expression of Fe-only nitrogenase.

### AlphaFold 3 predicts interactions between AnfA and deuridylylated P_II_ proteins

**Fig 4:**
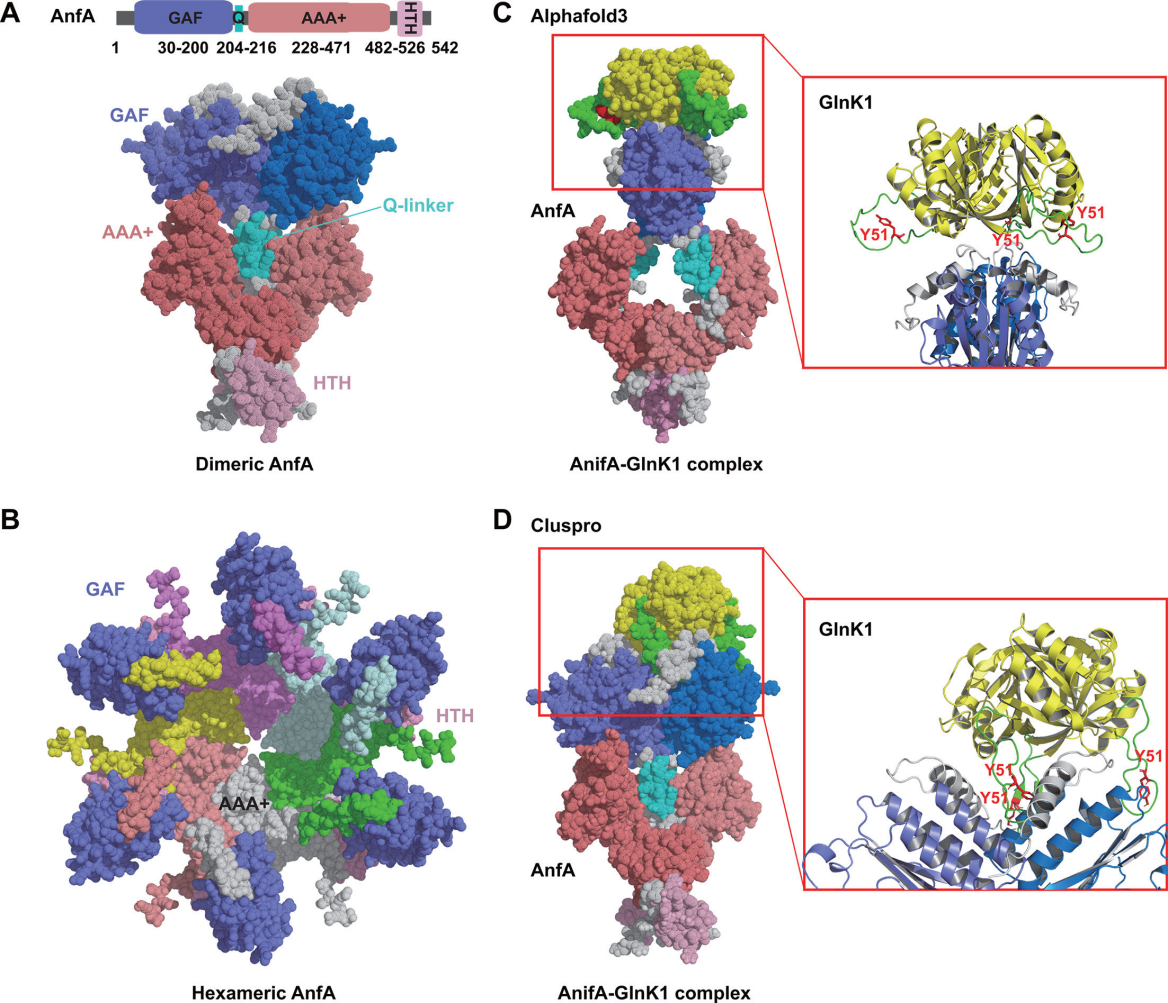
Interactions between AnfA and GlnK1 predicted by AlphaFold 3. (**A and B**) The predicted structures of dimeric and hexameric AnfA by AlphaFold 3. The top-ranked models for AnfA dimer (ipTM = 0.31, pTM = 0.42) and AnfA hexamer (ipTM = 0.40, pTM = 0.43) were used in the study. The GAF, Q-linker, AAA+, and HTH domains are colored in slate/marine, cyan, salmon/deep salmon, and pink/light pink, respectively. ipTM, interface predicted template modeling; pTM, predicted template modeling. (**C and D**) The docking model of AnfA-GlnK1 complex predicted by AlphaFold3 and Gluspro, respectively. The deuridylylated GlnK1 (yellow) shows potential interactions with the GAF domain of AnfA.

Oligomerization is a common feature among the family of bacterial enhancer-binding proteins (bEBPs), which are AAA+ proteins. As a member of the bEBP family, AnfA likely exists in two oligomeric states: an inactive dimer and an active hexamer (23–25). Consequently, both the dimeric and hexameric forms of AnfA were successfully modeled using AlphaFold 3. The protein modeling suggests that the dimeric AnfA displayed strong interactions among the GAF, AAA+ and HTH domains (Fig. 4A; Fig. S2), while the hexameric AnfA formed a ring structure via the AAA+ domains (Fig. 4B; Fig. S3). To explore potential interactions between P_II_ proteins and AnfA, we used AlphaFold 3 to predict docking between trimeric GlnK1 and dimeric AnfA. The model showed that the deuridylylated GlnK1 may interact with the GAF domain of AnfA, disrupting interaction between the AAA+ domains, which are crucial for the formation of the active hexameric AnfA (Fig. 4C). We further conducted rigid-body protein-protein docking simulations using ClusPro, indicating that GlnK1 interacts with one of the GAF domains of the AnfA dimer (Fig. 4D). These findings suggest that the deuridylylated P_II_ proteins may bind to the GAF domain of AnfA, hindering the conformational changes necessary for AnfA to form an active hexameric structure. The Uridylylated P_II_ proteins, whose uridine monophosphate (UMP) is located at the Tyr^51^ site on the T-loop, may alter the hydrogen bonding with the GAF domain, promoting the dissociation of P_II_ proteins and the formation of AnfA hexamer.

### The GAF domain of AnfA is potentially involved in interactions with deuridylylated GlnK1

**Fig 5:**
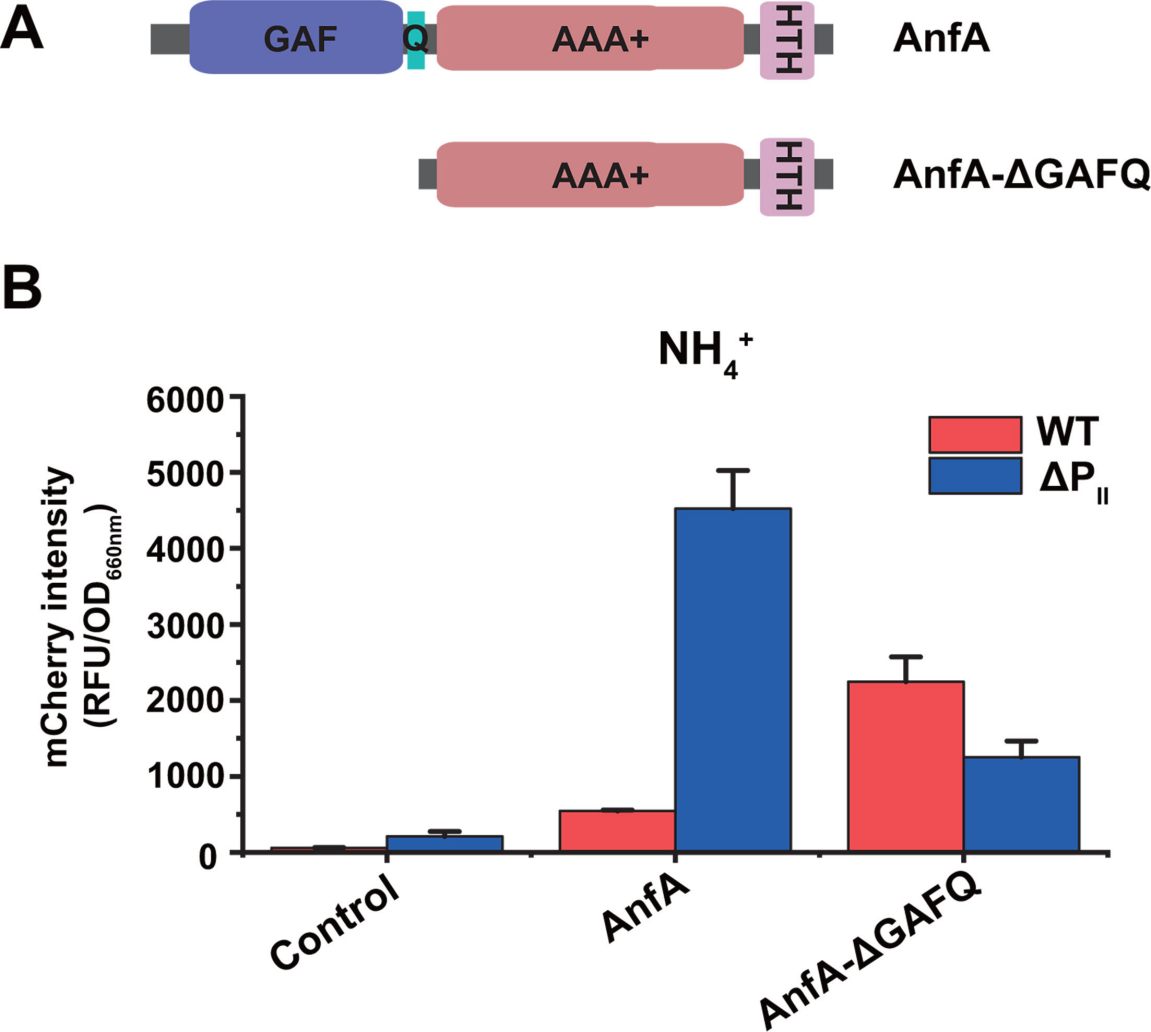
Deuridylylated GlnK1 interacts with the GAF domain of AnfA. (**A**) Schematic diagrams of the truncated AnfA lacking the GAF-Q domain. (**B**) Identification of the AnfA domain responsible for potential interactions with P_II_ proteins. The AnfA and its truncated variant lacking GAF/Q-linker domain (AnfA-ΔGAFQ) were expressed using the pBBR1MCS6-P*_anfH_*-RFP plasmid in *R. palustris* CGA009 (WT, red bar) and CGA3033 (ΔP_II_, blue bar), respectively. *R. palustris* CGA009 and CGA3033 harboring pBBR1MCS6-P*_nifH_*-RFP were used as negative controls. AnfA showed activity when its GAF-Q domain was removed. These data are the average of three independent experiments, and the error bars represent the SD.

To examine the role of the GAF domain in potential AnfA-P_II_ interactions, we expressed a truncated version of AnfA lacking the GAF-Q domains in both WT and ΔP_II_ strains (Fig. 5A). Both strains were grown with NH_4_^+^ under Mo-free conditions. The truncated AnfA lacking the GAF-Q domain showed transcriptional activation activity in both WT strain and ΔP_II_ mutant, suggesting that AnfA lacking the GAF-Q domain was no longer inhibited by deuridylylated P_II_ proteins (Fig. 5B). This suggests that deuridylylated P_II_ proteins may interact with the GAF domain of AnfA. Additionally, AlphaFold 3 modeling showed that AnfA lacking the GAF-Q domain could still form dimer and hexamer structures through its AAA+ domain (Fig. S4 and S5). These results demonstrate that the GAF domain may play a critical role in interacting with the deuridylylated P_II_ proteins.

## DISCUSSION

This study provides new insights into the regulation of Fe-only nitrogenase through AnfA. The transcriptional activator AnfA was previously believed to be regulated solely at the transcriptional level, with no knowledge of its post-translational regulation (5, 7). In *R. palustris* CGA009, the expression of *anfA* was significantly upregulated during diazotrophic growth with Fe-only nitrogenase in Mo-free medium, compared to growth with NH_4_^+^ under the same Mo-free conditions, thereby confirming its transcriptional regulation. This response aligns with the role of Fe-only nitrogenase in facilitating nitrogen fixation when fixed nitrogen and Mo are scarce. Similarly, this regulatory pattern is consistent with the transcriptional upregulation of the *nifA* gene when Mo is available for the expression of active Mo nitrogenase (14). However, while *nifA* exhibits basal expression under nitrogen-excess conditions, with only slight upregulation under nitrogen-fixing conditions, nearly no *anfA* expression was observed under nitrogen-excess conditions. Similarly, *Rhodobacter capsulatus* strains lacking all P_II_ proteins also failed to produce Fe-only nitrogenase in the presence of NH_4_^+^ (15). This indicates *anfA* transcription is triggered under conditions of more severe nitrogen deprivation than those required to initiate *nifA* transcription (9). Although the underlying mechanism remains unclear, it is speculated that increased levels of 2-oxoglutarate stimulate the transcription of the *anfA* gene (5).

Given that most of the P_II_ proteins sense cellular nitrogen status (2-oxoglutarate/glutamine) by binding 2-oxoglutarate, they may play a role in regulating the expression of the *anfA* gene (15, 26). As shown in Fig. 3C, uridylylated GlnK2, which is dramatically upregulated under nitrogen-fixing conditions (131.1-fold), is required to activate the transcription of the *anfA* gene. Under this condition, *R. palustris* primarily expresses the *glnK2* gene, with a TPM (Transcripts per million) value of 940.1, followed by the *glnB* gene (TPM, 211.6) and then the *glnK1* gene (TPM, 18.3). Consequently, the ΔP_II_ mutant produced less Fe-only nitrogenase than the WT CGA009 under nitrogen-fixing conditions (Fig. 3B). In addition to activating the transcription of the *nifA* gene, NtrC is also responsible for the transcriptional regulation of the *anfA* gene (10). Therefore, GlnK2 likely influences *anfA* transcription by modulating the phosphorylation status of NtrC (27). The binding of phosphorylated NtrC to the enhancer region is essential for the subsequent transcriptional activation of the *anf* gene cluster by AnfA. Besides transcriptional activation of *anfA*, the uridylylated GlnK2 also activates the transcription of *nifA* in *R. palustris* (14). Given that *R. palustris* preferentially expresses Mo nitrogenase, phosphorylated NtrC is expected to have a higher affinity for the *nifA* promoter than for the *anfA* promoter.

The absence of AnfA expression in the presence of NH_4_^+^ initially led to an oversight of the regulatory mechanisms controlling AnfA activity. No expression of Fe-only nitrogenase was detected in the presence of NH_4_^+^, even when AnfA was overexpressed in the WT CGA009 (Fig. 2B). Fe-only nitrogenase expression occurs only in strains lacking P_II_ proteins but expressing AnfA. Under this condition, *R. palustris* primarily expresses the *glnK1* gene, with a TPM value of 125.8, followed by the *glnB* gene (TMP, 25.6) and then the *glnK2* gene (TMP, 7.2). These pieces of evidence indicate that AnfA activity is still subject to post-translational regulation.

The formation of the NifA-P_II_ complex is known to repress NifA activity and nitrogenase expression in *R. palustris* (14). In *A. brasilense* and *Herbaspirillum seropedicae*, the binding of small molecules such as ATP and 2-oxoglutarate is required for P_II_ proteins to activate NifA (17, 28, 29). As members of the bEBPs family with a conserved AAA+ domain, AnfA and NifA likely share similar mechanisms for regulating their activity. Differential expression of P_II_ proteins was also observed between NH_4_^+^-grown and N_2_-grown *R. palustris* cells under Mo-free conditions (Fig. 1), suggesting that GlnK1, GlnK2, and GlnB may play distinct roles in regulating Fe-only nitrogenase. Overexpression of P_II_ proteins or P_II_^Y51F^ variants strongly represses AnfA activity in the presence of NH_4_^+^ (Fig. 3D). Given that *R. palustris* predominantly expresses GlnK1 under nitrogen-excess conditions, GlnK1 likely plays a major role in repressing AnfA activity. *R. palustris* ΔP_II_ mutant expressing GlnK1^Y51F^ was unable to grow under nitrogen-fixing conditions, suggesting that deuridylylated GlnK1 has a stronger inhibitory effect on AnfA activity. The inhibition of AnfA activity by GlnK1 may serve as a fail-safe mechanism, preventing *R. palustris* from expressing the Fe-only nitrogenase, even if AnfA is accidentally expressed under nitrogen-excess conditions. In contrast, the significantly upregulated GlnK2 under nitrogen-fixing conditions plays a major role in activating AnfA expression, likely through the NtrBC two-component system.

AnfA shares 36.22% amino acid sequence identity with NifA. Structure predictions of AnfA as a dimer or hexamer using AlphaFold3 indicate that AnfA oligomerizes through its AAA + domain, consistent with other typical AAA+ proteins (23). Deuridylylated GlnK1 interacts with NifA by fitting into the space between the GAF and the AAA + domains (14). Unlike NifA, AnfA adopts a more compact conformation with a short coil between its AAA+ and HTH domains. Interaction predictions between AnfA and deuridylylated GlnK1 using AlphaFold3 and Cluspro indicate that AnfA binds GlnK1 through its GAF domain.

The GAF domain is a universal receiver domain found across all kingdoms of life (30). In *R. palustris*, deuridylylated P_II_ proteins inhibit NifA activity and subsequently Mo nitrogenase expression by interacting with the GAF-AAA+ domains of NifA (14, 31, 32). Different from NifA, the truncated AnfA lacking the GAF-Q domain remains active regardless of the availability of NH_4_^+^ (Fig. 5). This finding indicates that AAA+ domain of AnfA may not be involved in interactions with deuridylylated P_II_ proteins. It is proposed that the binding of P_II_ proteins to AnfA dimer prevents the conformational transition required for AnfA to form an active hexamer. Deletion of the GAF-Q domain removes this inhibition, allowing AnfA to undergo conformational changes and assemble into an active AnfA hexamer capable of activating Fe-only nitrogenase expression via interaction with σ^54^ factor (33).

**Fig 6:**
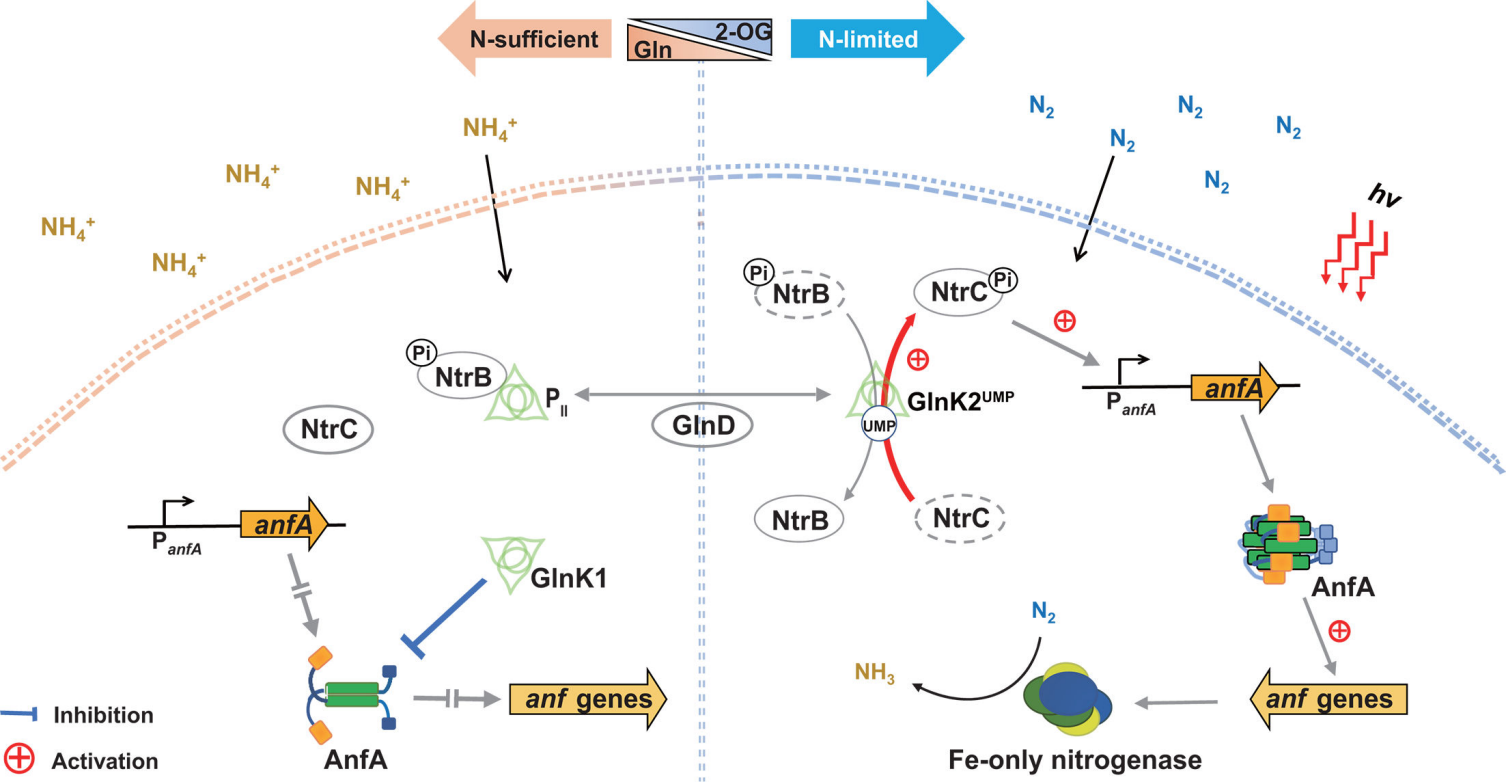
A model for the genetic regulation of Fe-only nitrogenase in response to nitrogen availability in *R. palustris*. Under nitrogen-replete (NH_4_^+^) conditions, no AnfA expression is observed. Additionally, AnfA activity can be inhibited by deuridylylated GlnK1, likely serving as a fail-safe mechanism. When *R. palustris* is exposed to nitrogen-limited (N_2_) and Mo-free conditions, uridylylated GlnK2 stimulates the expression of active AnfA, which further activates the expression of Fe-only nitrogenase.

In conclusion, this study uncovers the multi-layered regulatory mechanisms of Fe-only nitrogenase expression in response to nitrogen status. The transcriptional activator AnfA is regulated not only at the transcriptional level but also at the post-translational level by P_II_ proteins. Under nitrogen-fixing and Mo-free conditions, uridylylated GlnK2 significantly activates the transcription of AnfA through the NtrBC system. In contrast, under nitrogen-excess conditions, we hypothesize that the formation of an AnfA-GlnK1 complex can inhibit AnfA activity and subsequently Fe-only nitrogenase expression by preventing AnfA from forming an active hexamer. This likely acts as a safeguard mechanism to ensure that *R. palustris* does not express Fe-only nitrogenase in cases where AnfA is inadvertently activated under nitrogen-replete conditions (Fig. 6). A better understanding of Fe-only nitrogenase regulation will contribute to engineering diazotrophs for the development of more efficient nitrogen biofertilizers and subsequently promoting sustainable agriculture practices aimed at reducing soil degradation and carbon emissions.

## MATERIALS AND METHODS

### Bacterial strains and growth conditions

For photosynthetic growth, *R. palustris* strains were grown anaerobically in 27 mL sealed tubes containing 10 mL photosynthetic medium (PM) or nitrogen-fixing medium (NFM). NFM is identical to PM but lacks 7.5 mM ammonium sulfate. Mo was omitted from both PM and NFM as previously described (8). The tubes were incubated at 30°C under light with N₂ gas in the headspace. The PM and NFM used for growing cells were supplemented with 20 mM acetate as the carbon source. For genetic manipulation, *R. palustris* strains were grown aerobically on PM agar supplemented with 10 mM sodium succinate at 30°C (34). *Escherichia coli* S17-1 used for conjugation was grown in Luria-Bertani (LB) medium at 37°C. When appropriate, *R. palustris* and *E. coli* were grown with gentamicin at 100 and 20 µg mL^−1^, or with kanamycin at 200 and 50 µg mL^−1^.

### Genetic manipulation of *R. palustris*

All strains, plasmids, and primers used in this study are listed in Table S1. To construct the *R. palustris* CGA3047, a DNA sequence coding for eight histidines was introduced before the stop codon of *anfD*. The DNA fragment containing the DNA sequence for 8 × His tag and 1 kb of flanking DNA homologous to the chromosome of *R. palustris* was incorporated into *Bam*HI-digested pJQ-200SK suicide plasmid by the T5 exonuclease-dependent assembly method (35). The obtained pJQ-anfD-His was mobilized into *R. palustris* CGA3033 by conjugation with *E. coli* S17-1, and double-crossover events for allelic exchange were accomplished using a screening strategy as previously described (14). The plasmids carrying the mCherry reporter system were constructed using pBBR1MCS6 (14, 20, 36). The sequences were amplified as described in Table S1 and incorporated into pBBR1MCS6.

### Transcriptome analysis

The RNA-seq transcriptome library was constructed using total RNA following the user manual of Illumina TruSeq Stranded Total RNA Library Prep Kit and sequenced using the Illumina HiSeq X Ten system by Shanghai Majorbio Co. Ltd. (China). The high-quality clean reads selected by a Perl program were aligned to the genome by Bowtie2 and quantified by RSEM. The TPM (Transcripts Per Million reads) method was used to calculate the expression level. The differentially expressed genes (DEGs) were analyzed by DESeq2, and the false discovery rate in multiple hypothesis testing was corrected by the “Benjamini/Hochberg” method with parameters: *P* adjust <0.05 and |log_2_FC| ≥ 1. A volcano plot of the DEGs was drawn by the “ggplot2” R package.

### Proteome analysis

Proteome sequencing was performed by Shanghai Majorbio Co. Ltd. (China). The protein extracted from cells was treated with trypsin and labeled by TMT reagents. The TMT-labeled peptide samples in each group were mixed in a 1:1 ratio, then vacuum concentrated to almost dryness and re-solubilized with UPLC loading buffer (2% acetonitrile, pH 10). The peptides were separated by a reversed-phase C18 column (1.7 µm, 2.1 mm × 150 mm) and analyzed by Easy-nLC 1200 liquid chromatography pump coupled with a Q Exactive HF-X mass spectrometer. The clean data were analyzed using the free online Majorbio Cloud Platform. Differentially expressed proteins (DEPs) were identified based on a fold change (≥1.5 or ≤0.67) and *P* value < 0.05.

### Fluorescence intensity measurement

The fluorescence intensity was detected according to the protocol described previously (20). Cells grown to the mid-logarithmic phase were harvested and washed two times with 10 mM phosphate-buffered saline (PBS, pH 7.2), then resuspended with 500 µL PBS. Aliquots of 200 µL samples were measured for OD_660_ and relative fluorescence by using clear and black 96-well microplates after being exposed to air for 4 h, respectively. The excitation and emission wavelengths for the relative fluorescence measurement were set to 587 and 610 nm.

### Nitrogenase activity measurement

Nitrogenase activity of *R. palustris* was determined by measuring the H_2_ and CH_4_ production and acetylene reduction assay (14). The gas-phase samples (100 µL) for H_2_ and CH_4_ determination were withdrawn from the headspace of sealed tubes when *R. palustris* grew to the stationary phase. For the acetylene reduction assay, 4 mL cultures that reached the mid-logarithmic phase was injected into the 27 mL sealed tubes filled with argon gas and added 2% C_2_H_2_ (final concentration) to initiate the assay at 30°C in light. The gas samples (100 µL) were withdrawn from the headspace of the sealed tubes at intervals. H_2_ and CH_4_/C_2_H_6_ were measured with a Shimadzu GC-2014 gas chromatograph with the 60/80 molecular sieve 5A column (1.8 m × 1/8 in. × 2.1 mm i.d.) and 80/100 Porapak N column (1.8 m × 1/8 in. × 2.1 mm i.d.). The columns' temperature was held at 60°C, the thermal conductivity detector (TCD) and its injector temperatures were set to 120°C and 85°C, the flame ionization detector (FID) and its injector temperatures were set to 150°C and 120°C, respectively. The rate of carrier gas argon was 25 mL/min (37, 38).

### Quantitative real-time PCR (qRT-PCR)

qRT-PCR was used to measure the expression levels of the *anfA* gene of *R. palustris* strains in mid-logarithmic phase. The cell pellets were collected with centrifugation and flash frozen by liquid nitrogen. RNA was extracted and reverse transcribed to cDNA sequentially using RNAsimple Total RNA Kit (TIANGEN, China) and FastKing-RT SuperMix (TIANGEN, China). qRT-PCR was carried out on ABI 7500 PCR system (Applied Biosystems, USA) using SuperReal PreMix Plus (TIANGEN, China) with 40 cycles of 95°C for 10 s, 60°C for 20 s, and 72°C for 32 s. The housekeeping gene *rpoD* was used as the endogenous reference gene.

### Western blotting

The cultures grown in PM were harvested by centrifugation at 12,000 × *g* for 1 min at room temperature and resuspended in buffer (20 mM Tris-HCl [pH 8.0], 300 mM NaCl). The total protein of cells was extracted by sonication (36), and concentrations of protein were measured by the Bradford method. Around 30 µg of protein was loaded per SDS-PAGE well. Proteins were transferred to polyvinylidene difluoride (PVDF) membrane. The prestained Blue Plus II Protein Marker (14–120 kDa) (TransGen Biotech, China) was used to indicate the electrophoresis and electrophoretic transfer. A mouse monoclonal antibody against the His-tag and an HRP-labeled Goat Anti-Mouse IgG (H + L) were used as primary and conjugated antibodies, respectively. The PVDF membrane was washed and detected by the BeyoECL Moon reagent (Beyotime, Shanghai, China) using a chemiluminescence image analyzer (Tanon, Shanghai, China).

### Structural modeling and docking

The structures of the oligomeric AnfA and GlnK1 were predicted using the AlphaFold3 online server (https://golgi.sandbox.google.com/) (39). AnfA dimer: seed number was set to 10. The model (ipTM = 0.31 and pTM = 0.42) is used throughout the text. AnfA hexamer:seed number was set to 10. The top-ranked model (ipTM = 0.40 and pTM = 0.43) is used throughout the text. GlnK1 trimer:seed number was set to 10. The model (ipTM = 0.88 and pTM = 0.89) is used throughout the text. AnfA-GlnK1 complex:seed number was set to 10. The model (ipTM = 0.27 and pTM = 0.33) is used throughout the text. AnfA-ΔGAFQ dimer:seed number was set to 10. The model (ipTM = 0.15 and pTM = 0.45) is used throughout the text. AnfA-ΔGAFQ hexamer:seed number was set to 10. The top-ranked model (ipTM = 0.62 and pTM = 0.66) is used throughout the text. ClusPro 2.0 (https://cluspro.bu.edu/) was used for the docking of trimeric GlnK1 to dimeric AnfA. The top-ranked model by ClusPro with the largest cluster size based on the minimized interaction energy (top member of balanced [weighted score of center = −1,093.4, weighted score of lowest energy = −1,176] = 47, top member of Electrostatic-favored [weighted score of center = −1,191.5, weighted score of lowest energy = −1,191.5] = 41, top member of Hydrophobic-favored [weighted score of center = −1,590.8, weighted score of lowest energy = −1,590.8] = 46, top member of van der Waals [vdW] energy + electrostatic energy [weighted score of center = −241.2, weighted score of lowest energy = −270.1] = 53) is used in this paper. PyMOL was used to visualize the three-dimensional models of the proteins.

## Data Availability

Transcriptome data are publicly available at the NCBI Sequence Read Archive under the accession number PRJNA937073, and proteomics data are publicly available at iProX database under the accession number PXD042610.

## SUPPLEMENTAL MATERIAL

Supplemental material aem.00465-25-s0001.docx Strains, plasmids, primers, phenotypes, and predicted structures of AnfA and its variant.

ASM does not own the copyrights to Supplemental Material that may be linked to, or accessed through, an article. The authors have granted ASM a non-exclusive, world-wide license to publish the Supplemental Material files. Please contact the corresponding author directly for reuse.
