# Supplementary material for: PII protein is essential for transcriptional regulation of anf gene cluster for iron-only nitrogenase in Rhodopseudomonas palustris
Source: Appl Environ Microbiol. 2025 Apr 10;91(5):e00465-25. doi: 10.1128/aem.00465-25 (PMC12093980; doi:10.1128/aem.00465-25)
Supplement: Supplemental material — Strains, plasmids, primers, phenotypes, and predicted structures of AnfA and its variant. [file aem.00465-25-s0001.docx]

**SUPPORTING INFORMATION FOR**

P_II_ protein is essential for transcriptional regulation of *anf* gene cluster for iron-only nitrogenase

Yan Zeng^1,#^, Lingwei Cui^1,2,#^, Mengmei Wang^1,2^, Lu Huang^1,2^, Mingyue Jiang^1,2^, Ying Liu^1^, Yongqiang Gao^3^ and Yanning Zheng^1^*

^1^ State Key Laboratory of Microbial Resources, Institute of Microbiology, Chinese Academy of Sciences, Beijing, China.

^2^ College of Life Sciences, University of Chinese Academy of Sciences, Beijing, China.

^3^ Department of Microbiology, Harvard Medical School, Boston, MA 02115, USA

^#^ These authors contributed equally to this work.

* Corresponding author. Corresponding author. Mailing address: No.1 Beichen West Road, Chaoyang District, Beijing 100101, China. Tel: +86-10-64806943. E-mail: zhengyn@im.ac.cn

**This file includes:**

Table S1.

Figures S1-S5.

**Table S1.** Strains and plasmids used in this study.

| **Strain or plasmid** | **Genotype or phenotype** | **Source** |
| --- | --- | --- |
| ***R. palustris* strains** |  |  |
| CGA009 | Wild type; *hupV* mutant; spontaneous frameshift (4-bp deletion) in *hupV* | 1 |
| CGA676 | Derived from CGA757; *nifA**; 48 bp deletion encoding Q-linker amino acids 202-217; expressis Mo-nitrogenase in the presence of NH_4_^+^ | 2 |
| CGA3020 | CGA009 NifA* AnfD-His ΔP*_anfH_*::P*_nifH_* | 3 |
| CGA3033 | CGA009 *ΔglnK1-amtB1-glnK2-amtB2 ΔglnB::kan* | 4 |
| CGA3047 | CGA3033 AnfD-His | This study |
| CGA009-7 | CGA009 harboring plasmid pBBR1MCS6-P*_anfH_*-RFP | This study |
| CGA009-8 | CGA009 harboring plasmid pBBR1MCS6-P*_J23119_*-AnfA-P*_anfH_*-RFP | This study |
| CGA009-9 | CGA009 harboring plasmid pBBR1MCS6-PJ23119-AnfA-dGAFQ-P*_anfH_*-RFP | This study |
| CGA3033-13 | CGA3033 harboring plasmid pBBR1MCS6-P*_anfH_*-RFP | This study |
| CGA3033-14 | CGA3033 harboring plasmid pBBR1MCS6-P*_J23119_*-AnfA-P*_anfH_*-RFP | This study |
| CGA3033-15 | CGA3033 harboring plasmid pBBR1MCS6-P*_anfH_*-RFP-P*_GAPDH_*-GlnK1 | This study |
| CGA3033-16 | CGA3033 harboring plasmid pBBR1MCS6-P*_anfH_*-RFP-P*_GAPDH_*-GlnK2 | This study |
| CGA3033-17 | CGA3033 harboring plasmid pBBR1MCS6-P*_anfH_*-RFP-P*_GAPDH_*-GlnB | This study |
| CGA3033-18 | CGA3033 harboring plasmid pBBR1MCS6-P*_J23119_*-AnfA-P*_anfH_*-RFP-P*_GAPDH_*-GlnK1 | This study |
| CGA3033-19 | CGA3033 harboring plasmid pBBR1MCS6-P*_J23119_*-AnfA-P*_anfH_*-RFP-P*_GAPDH_*-GlnK2 | This study |
| CGA3033-20 | CGA3033 harboring plasmid pBBR1MCS6-P*_J23119_*-AnfA-P*_anfH_*-RFP-P*_GAPDH_*-GlnB | This study |
| CGA3033-21 | CGA3033 harboring plasmid pBBR1MCS6-P*_J23119_*-AnfA-P*_anfH_*-RFP-P*_GAPDH_*-GlnK1-Y51F | This study |
| CGA3033-22 | CGA3033 harboring plasmid pBBR1MCS6-P*_J23119_*-AnfA-P*_anfH_*-RFP-P*_GAPDH_*-GlnK2-Y51F | This study |
| CGA3033-23 | CGA3033 harboring plasmid pBBR1MCS6-P*_J23119_*-AnfA-P*_anfH_*-RFP-P*_GAPDH_*-GlnB-Y51F | This study |
| CGA3033-24 | CGA3033 harboring plasmid pBBR1MCS6-PJ23119-AnfA-dGAFQ-P*_anfH_*-RFP | This study |
| ***E. coli* strain** |  |  |
| S17-1 | *thi pro hdsR hdsM^+^ recA*; chromosomal insertion of RP4-2 (Tc::Mu Km::Tn7) | 5 |
| **Plasmids** |  |  |
| pJQ-200SK | Gm^R^, *sacB*; mobilizable suicide vector | 6 |
| pJQ-anfD-His | Gm^R^, His8-tag was inserted before the stop codon of anfD | This study |
| pBBR1MCS6 | Gm^R^; T7 terminator inserted into *Kpn*I site of pBBR1MCS5 | 3 |
| pBBR1MCS6-P*_anfH_*-RFP | Gm^R^; promotor of *anfH* and gene *mcherry* inserted into *Hin*dIII site of pBBR1MCS6 | 3 |
| pBBR1MCS6-P*_J23119_*-anfA-P*_anfH_*-RFP | Gm^R^; promotor of J23119 and gene *anfA* inserted into *Bam*HI site of pBBR1MCS6-P*_anfH_*-RFP | This study |
| pBBR1MCS6-P*_anfH_*-RFP-P*_GAPDH_*-GlnK1 | Gm^R^; promotor of *anfH* and gene *mcherry* inserted into *Eco*RI/*Hin*dIII site of pBBR1MCS6, then the promoter P*_GAPDH_* and gene *glnK1* cloned from pBBR1MCS6-P*_nifH_*-RFP-P*_GAPDH_*-GlnK1 were inserted into the *Kpn*I site | This study  4 |
| pBBR1MCS6-P*_anfH_*-RFP-P*_GAPDH_*-GlnK2 | Gm^R^; promotor of *anfH* and gene *mcherry* inserted into *Eco*RI/*Hin*dIII site of pBBR1MCS6, then the promoter P*_GAPDH_* and gene *glnK2* cloned from pBBR1MCS6-P*_nifH_*-RFP-P*_GAPDH_*-GlnK2 were inserted into the *Kpn*I site | This study  4 |
| pBBR1MCS6-P*_anfH_*-RFP-P*_GAPDH_*-GlnB | Gm^R^; promotor of *anfH* and gene *mcherry* inserted into *Eco*RI/*Hin*dIII site of pBBR1MCS6, then the promoter P*_GAPDH_* and gene *glnB* cloned from pBBR1MCS6-P*_nifH_*-RFP-P*_GAPDH_*-GlnB were inserted into the *Kpn*I site | This study  4 |
| pBBR1MCS6-P*_J23119_*-anfA-P*_anfH_*-RFP-P*_GAPDH_*-GlnK1 | Gm^R^; the promoter P*_GAPDH_* and gene *glnK1* cloned from pBBR1MCS6-P*_nifH_*-RFP-P*_GAPDH_*-GlnK1 were inserted into the *Kpn*I site of pBBR1MCS6- P*_J23119_*-anfA-P*_anfH_*-RFP | This study  4 |
| pBBR1MCS6-P*_J23119_*-anfA-P*_anfH_*-RFP-P*_GAPDH_*-GlnK2 | Gm^R^; the promoter P*_GAPDH_* and gene *glnK2* cloned from pBBR1MCS6-P*_nifH_*-RFP-P*_GAPDH_*-GlnK2 were inserted into the *Kpn*I site of pBBR1MCS6-P*_J23119_*-anfA-P*_anfH_*-RFP | This study  4 |
| pBBR1MCS6-P*_J23119_*-anfA-P*_anfH_*-RFP-P*_GAPDH_*-GlnB | Gm^R^; the promoter P*_GAPDH_* and gene *glnB* cloned from pBBR1MCS6-P*_nifH_*-RFP-P*_GAPDH_*-GlnB were inserted into the *Kpn*I site of pBBR1MCS6-P*_J23119_*-anfA-P*_anfH_*-RFP | This study  4 |
| pBBR1MCS6-P*_J23119_*-anfA-P*_anfH_*-RFP-P*_GAPDH_*-GlnK1-Y51F | Gm^R^; *glnK1*-*Y51F*: A_152_ was replaced by T by overlap extension PCR of pBBR1MCS6-P*_J23119_*-anfA-P*_anfH_*-RFP-P*_GAPDH_*-GlnK1. | This study |
| pBBR1MCS6-P*_J23119_*-anfA-P*_anfH_*-RFP-P*_GAPDH_*-GlnK2-Y51F | Gm^R^; *glnK2*-*Y51F*: A_152_ was replaced by T by overlap extension PCR of pBBR1MCS6-P*_J23119_*-anfA-P*_anfH_*-RFP-P*_GAPDH_*-GlnK2. | This study |
| pBBR1MCS6-P*_J23119_*-anfA-P*_anfH_*-RFP-P*_GAPDH_*-GlnB-Y51F | Gm^R^; *glnB*-*Y51F*: A_152_ was replaced by T by overlap extension PCR of pBBR1MCS6-P*_J23119_*-anfA-P*_anfH_*-RFP-P*_GAPDH_*-GlnB. | This study |
| pBBR1MCS6-PJ23119-anfA-dGAFQ-P*_anfH_*-RFP | GmR; Gm^R^; the *anfA* gene was cut from pBBR1MCS6-P*_J23119_*-anfA-P*_anfH_*-RFP and truncated gene *anfA-dGAFQ* inserted into *Eco*RI site, AnfA-dGAFQ: 645 bp deletion encoding GAF amino acids 2-216. | This study |
| **Primers** |  |  |
| anfD-His-up-F | GCGGCCGCTCTAGAACTAGTCACCACAAGATTAACATCGCC | For construction of pJQ-anfD-His |
| anfD-His-up-R | GGCGTGGTGGTGGTGGTGGTGGTGGTGCGCTTCGGCTTTGACGATC |  |
| anfD-His-down-F | GCGCACCACCACCACCACCACCACCACGCCGAGTGAGACGAGAGC |  |
| anfD-His-down-R | TCCTGCAGCCCGGGGGATCCCTGTGATCAGATTGATCTTCAGGC |  |
| B6-anfA-up-F | GCTCTAGAACTAGTGGATCCTTGACAGCTAGCTCAGTCCTAGGTATAATACTAGTAAGGAGATATACATGAATTCATGACGTCGCTCGAGCTGAC | For construction of pBBR1MCS6-P*_J23119_*-anfA-P*_anfH_*-RFP |
| B6-anfA-up-R | AATTCCTGCAGCCCGGGGTCAGTTCCGCGGGCGCAG |  |
| B6-PanfH-RFP-down-F | ATTCGATATCAAGCTTTTTTGCGTTGGCGGAGAGCTC |  |
| B6-PanfH-RFP-down-R | GAGGTCGACGGTATCGATTTAGCCGGCCTTGTACAGCTCG |  |
| P*_anfH_*-P_II_-F | GTGGATCCCCCGGGCTGCAGGAATTCTTTTGCGTTGGCGG | For construction of pBBR1MCS6-P*_anfH_*-RFP-P*_GAPDH_*-GlnK1 |
| P*_anfH_*-P_II_-R | TCGAGGTCGACGGTATCGATTTAGCCGGCCTTGTACAGC |  |
| B6-anfA-PG-F | TCGACCTCGAGGGGGGGCCCATATCTCGGCGCCCACACTC |  |
| B6-nifA-PG-K1-R | CTTTCGGGCTTTGGGTACCTTACAGCGCGTCGACGTCGG |  |
| P*_anfH_*-P_II_-F | GTGGATCCCCCGGGCTGCAGGAATTCTTTTGCGTTGGCGG | For construction of pBBR1MCS6-P*_anfH_*-RFP-P*_GAPDH_*-GlnK2 |
| P*_anfH_*-P_II_-R | TCGAGGTCGACGGTATCGATTTAGCCGGCCTTGTACAGC |  |
| B6-anfA-PG-F | TCGACCTCGAGGGGGGGCCCATATCTCGGCGCCCACACTC |  |
| B6-nifA-PG-K2-R | CTTTCGGGCTTTGGGTACCTCAGAGGGCGGCCGCGTC |  |
| P*_anfH_*-P_II_-F | GTGGATCCCCCGGGCTGCAGGAATTCTTTTGCGTTGGCGG | For construction of pBBR1MCS6-P*_anfH_*-RFP-P*_GAPDH_*-GlnB |
| P*_anfH_*-P_II_-R | TCGAGGTCGACGGTATCGATTTAGCCGGCCTTGTACAGC |  |
| B6-anfA-PG-F | TCGACCTCGAGGGGGGGCCCATATCTCGGCGCCCACACTC |  |
| B6-nifA-PG-B-R | CCTTTCGGGCTTTGGGTACCTTAGATGGCGTCCAGTCCGG |  |
| B6-anfA-PG-F | TCGACCTCGAGGGGGGGCCCATATCTCGGCGCCCACACTC | For construction of pBBR1MCS6-P*_J23119_*-anfA-P*_anfH_*-RFP-P*_GAPDH_*-GlnK1 |
| B6-nifA-PG-K1-R | CTTTCGGGCTTTGGGTACCTTACAGCGCGTCGACGTCGG |  |
| B6-anfA-PG-F | TCGACCTCGAGGGGGGGCCCATATCTCGGCGCCCACACTC | For construction of pBBR1MCS6-P*_J23119_*-anfA-P*_anfH_*-RFP-P*_GAPDH_*-GlnK2 |
| B6-nifA-PG-K2-R | CTTTCGGGCTTTGGGTACCTCAGAGGGCGGCCGCGTC |  |
| B6-anfA-PG-F | TCGACCTCGAGGGGGGGCCCATATCTCGGCGCCCACACTC | For construction of pBBR1MCS6-P*_J23119_*-anfA-P*_anfH_*-RFP-P*_GAPDH_*-GlnB |
| B6-nifA-PG-B-R | CCTTTCGGGCTTTGGGTACCTTAGATGGCGTCCAGTCCGG |  |
| GlnK1-Y51F-F | GTGGCGCCGAGTTCATCGTGAATTTCCTGCCCAAGCTGCGG | 4 |
| GlnK1-Y51F-R | GAAATTCACGATGAACTCGGCGCCACGATAGATCTCGGTATGGCC |  |
| GlnK2-Y51F-F | GCGGCGCTGAATTCGCGGTGAGCTTCCTGCCCAAGATC |  |
| GlnK2-Y51F-R | GGAAGCTCACCGCGAATTCAGCGCCGCGGTAGATTTCC |  |
| GlnB-Y51F-F | GCGGCGCTGAATTCATCGTCGACTTCCTGCCCAAGGTGAAAATC |  |
| GlnB-Y51F-R | GTCGACGATGAATTCAGCGCCGCGATACAACTCGGCGTGG |  |
| pB6-anfA-dGAFQ-F | GAATTCATGCGCTTTCGCCCGACCAA | For construction of pBBR1MCS6-P*_J23119_*-anfA-dGAFQ-P*_anfH_*-RFP |
| B6-anfA-up-R | AATTCCTGCAGCCCGGGGTCAGTTCCGCGGGCGCAG |  |
| Q-anfA-F | TCGAGAACGTCGACAAGGTG | For qPCR |
| Q-anfA-R | GTTGCCGATGATGTTGGTCG |  |

#
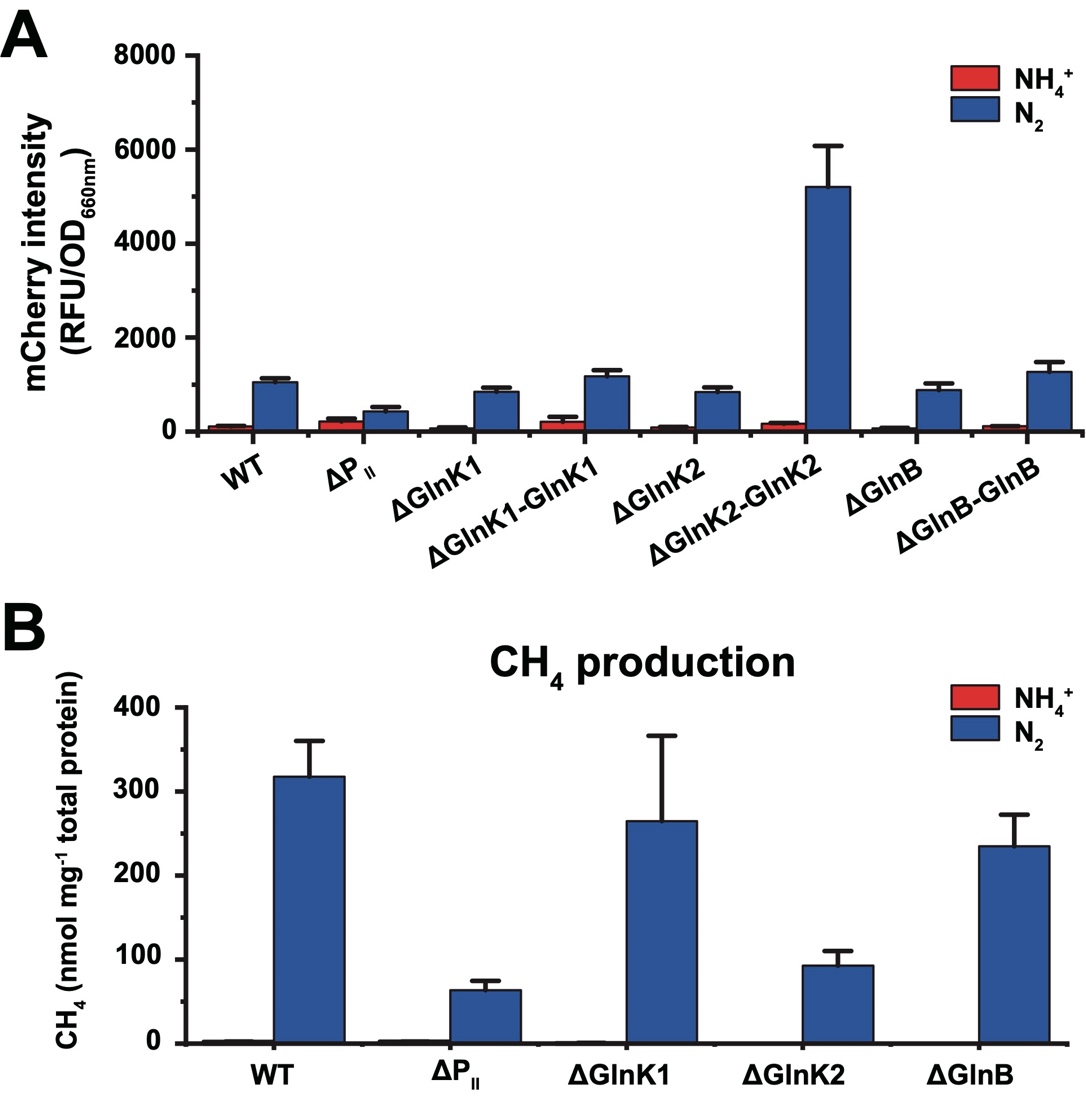


# Fig. S1 GlnK2 stimulates the expression of active Fe-only nitrogenase under nitrogen-fixing conditions. (A) The influence of P_II_ proteins (GlnK1, GlnK2 and GlnB) on the expression of Fe-only nitrogenase. (B) CH_4_ production was determined by gas-chromatography to examine the activity of Fe-only nitrogenase. These data are the average of three independent experiments, and the error bars represent the SD.


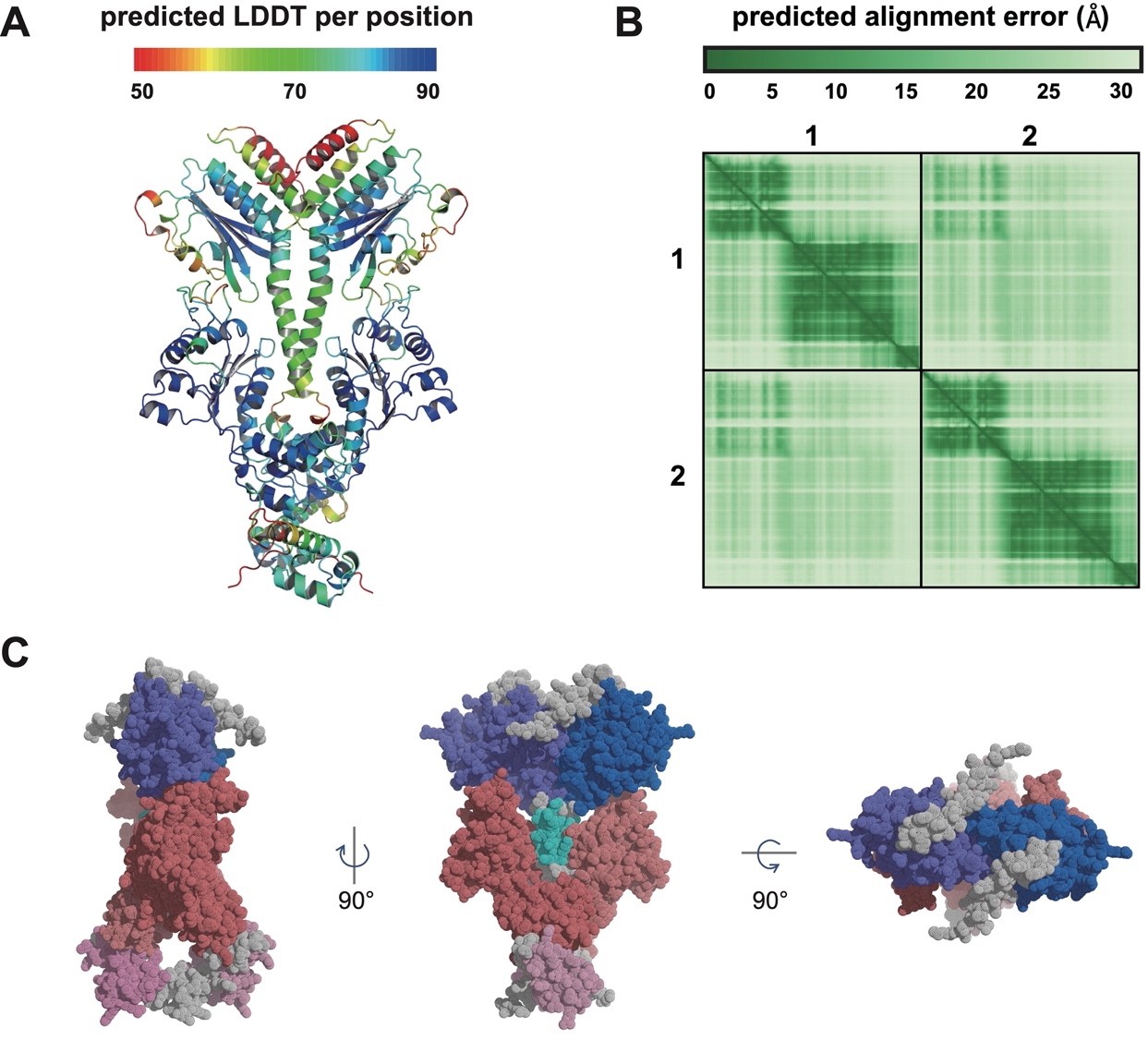


# Fig. S2 Predicted structure of the dimeric AnfA by Alphafold3. (A) Predicted local distance difference tests (pLDDT) per position mapped onto the AnfA dimer model. (B) Predicted alignment error (PAE) in Å of all residues against all residues for the top-ranked model. (C) The three views show the structure of dimeric AnfA. The GAF, Q-linker, AAA+ and HTH domains are colored in slate/marine, cyan/pale cyan, salmon/deep salmon, and pink/light pink, respectively.


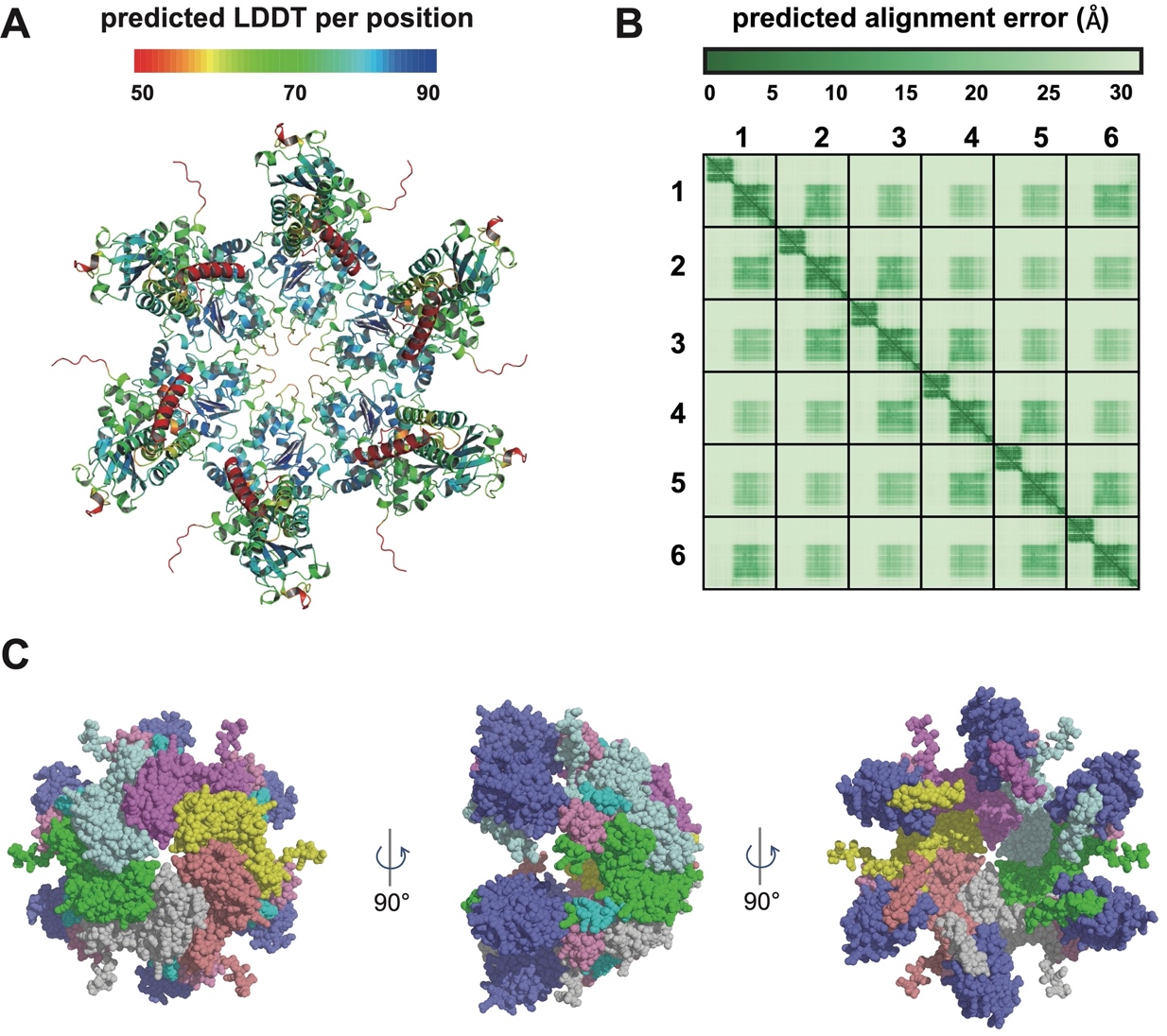


# Fig. S3 Predicted structure of the hexameric AnfA by Alphafold3. (A) Predicted local distance difference tests (pLDDT) per position mapped onto the AnfA hexamer model. (B) Predicted alignment error (PAE) in Å of all residues against all residues for the top-ranked model. (C) The three views show the structure of hexameric AnifA. The GAF, Q-linker, AAA+ and HTH domains are colored in slate/marine, cyan, yellow/violet/pale cyan/green/gray/salmon, and pink, respectively.


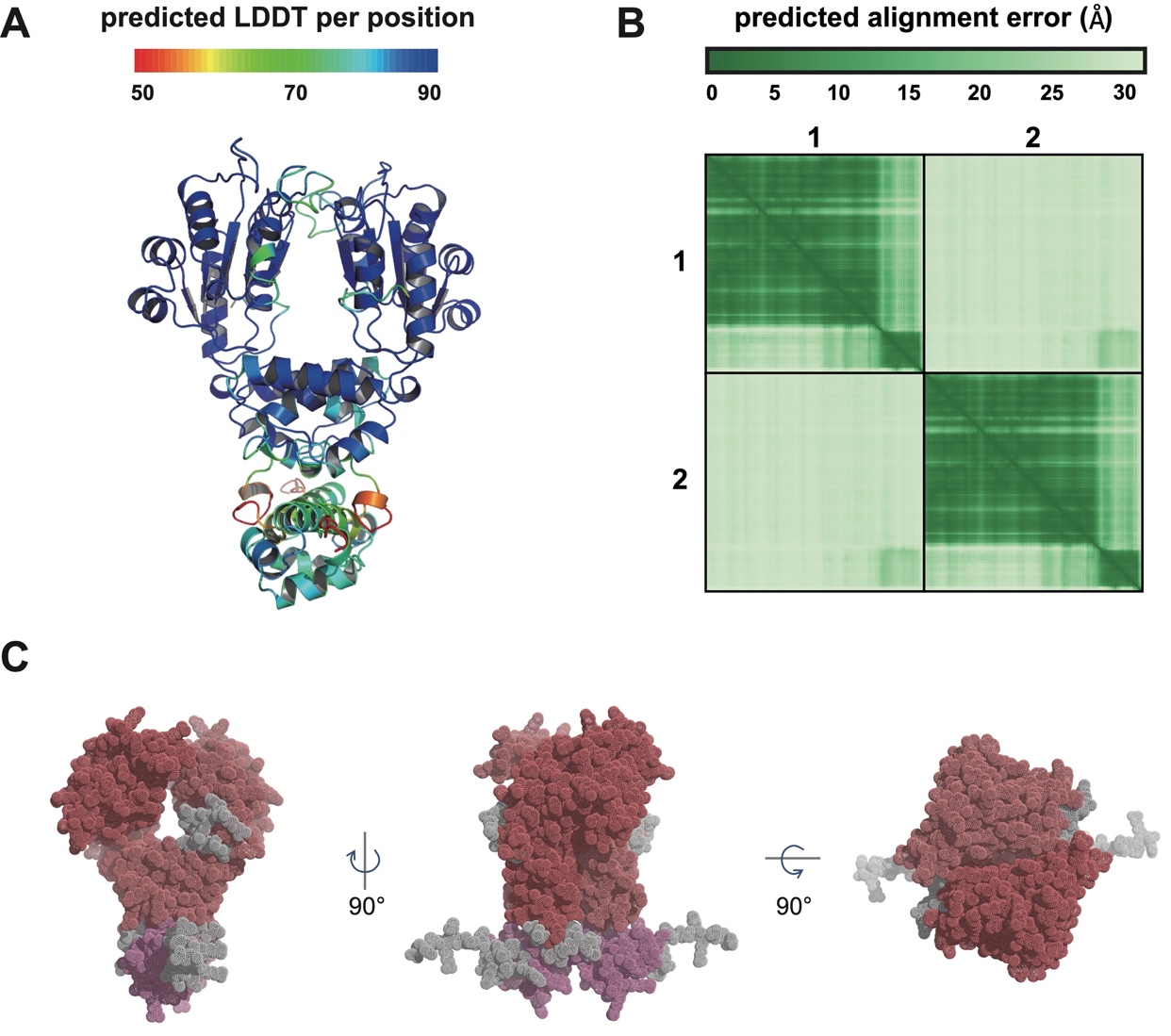


# Fig. S4 Predicted structure of the dimeric AnfA-ΔGAFQ by Alphafold3. (A) Predicted local distance difference tests (pLDDT) per position mapped onto the AnfA-ΔGAFQ dimer model. (B) Predicted alignment error (PAE) in Å of all residues against all residues for the top-ranked model. (C) The three views show the structure of dimeric AnfA-ΔGAFQ. The AAA+ and HTH domains are colored in salmon/deep salmon, and pink/light pink, respectively.


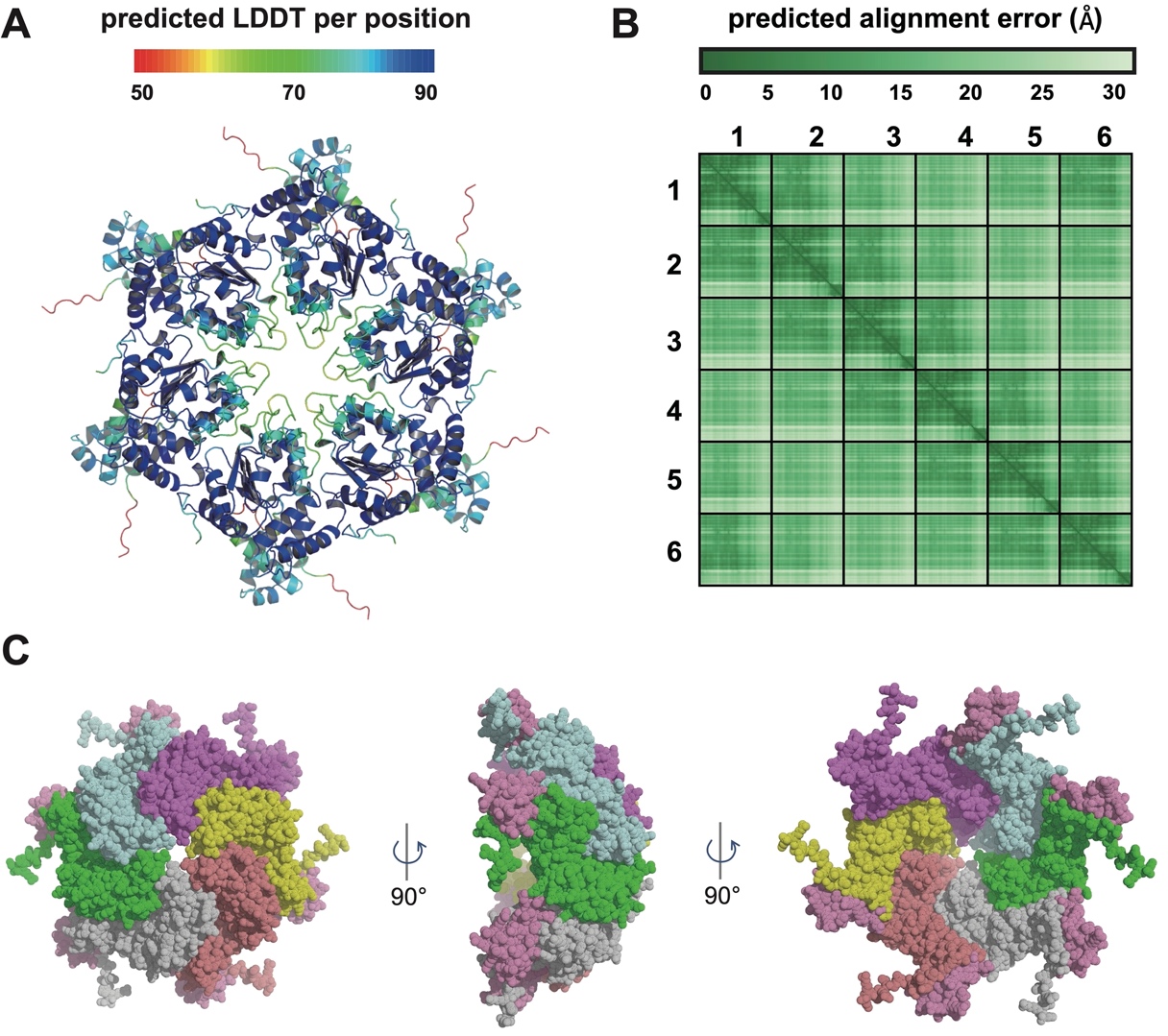


# Fig. S5 Predicted structure of the hexameric AnfA-ΔGAFQ by Alphafold3. (A) Predicted local distance difference tests (pLDDT) per position mapped onto the AnfA-ΔGAFQ hexamer model. (B) Predicted alignment error (PAE) in Å of all residues against all residues for the top-ranked model. (C)The three views show the structure of hexameric AnfA-ΔGAFQ. The AAA+ and HTH domains are colored in yellow/violet/pale cyan/green/gray/salmon, and pink, respectively.

**REFERENCES**

1. Larimer FW, Chain P, Hauser L, Lamerdin J, Malfatti S, Do L, Land ML, Pelletier DA, Beatty JT, Lang AS, Tabita FR, Gibson JL, Hanson TE, Bobst C, Torres JL, Peres C, Harrison FH, Gibson J, Harwood CS. 2004. Complete genome sequence of the metabolically versatile photosynthetic bacterium *Rhodopseudomonas palustris*. *Nat Biotechnol* 22:55-61.

2. James BM, Caroline SH. 2010. Carbon dioxide fixation as a central redox cofactor recycling mechanism in bacteria. *Proc Natl Acad Sci USA* 107:11669-11675.

3. Zeng Y, Wang M, Yu Y, Wang L, Cui L, Li C, Liu Y, Zheng Y. 2024. Rice N-biofertilization by inoculation with an engineered photosynthetic diazotroph. *World J Microbiol Biotechnol* 40:136.

4. Zeng Y, Guo L, Gao Y, Cui L, Wang M, Huang L, Jiang M, Liu Y, Zhu Y, Xiang H, Li D-F, Zheng Y. 2024. Formation of NifA-P_II_ complex represses ammonium-sensitive nitrogen fixation in diazotrophic proteobacteria lacking NifL. *Cell Rep* 43:114476.

5. Simon R, Priefer U, Pühler A. 1983. A broad host mobilization system for in vivo genetic engineering: transposon mutagenesis in Gram-negative bacteria. *Nat Biotechnol* 1:784–791.

6. Quandt J, Hynes MF. 1993. Versatile suicide vectors which allow direct selection for gene replacement in Gram-negative bacteria. *Gene* 127:15-21.
